# Pharmacokinetics of Jaspine B and Enhancement of Intestinal Absorption of Jaspine B in the Presence of Bile Acid in Rats

**DOI:** 10.3390/md15090279

**Published:** 2017-09-01

**Authors:** Min-Koo Choi, Jihoon Lee, So Jeong Nam, Yun Ju Kang, Youjin Han, Kwangik Choi, Young A. Choi, Mihwa Kwon, Dongjoo Lee, Im-Sook Song

**Affiliations:** 1 College of Pharmacy, Dankook University, Cheon-an 31116, Korea; minkoochoi@dankook.ac.kr (M.-K.C.); ayha06@gmail.com (Y.A.C.); 2 College of Pharmacy and Research Institute of Pharmaceutical Sciences, Kyungpook National University, Daegu 41566, Korea; legadema0905@naver.com (J.L.); goddns159@nate.com (S.J.N.); yun-ju6895@nate.com (Y.J.K.); gksdbwls2@nate.com (Y.H.); reggirchoi@naver.com (K.C.); mihwa_k@naver.com (M.K.); 3 College of Pharmacy, Ajou University, Suwon 16499, Korea; dongjoo@ajou.ac.kr

**Keywords:** Jaspine B, bile salts, intestinal permeability, bioavailability, metabolic instability

## Abstract

We aimed to investigate the pharmacokinetics and the underlying mechanisms of the intestinal absorption, distribution, metabolism, and excretion of Jaspine B in rats. The oral bioavailability of Jaspine B was 6.2%, but it decreased to 1.6% in bile-depleted rats and increased to 41.2% (normal) and 23.5% (bile-depleted) with taurocholate supplementation (60 mg/kg). Consistent with the increased absorption in the presence of bile salts, rat intestinal permeability of Jaspine B also increased in the presence of 10 mM taurocholate or 20% bile. Further studies demonstrated that the enhanced intestinal permeability with bile salts was due to increased lipophilicity and decreased membrane integrity. Jaspine B was designated as a highly tissue-distributed compound, because it showed large tissue to plasma ratios in the brain, kidney, heart, and spleen. Moreover, the recovery of Jaspine B from the feces and urine after an intravenous administration was about 6.3%, suggesting a substantial metabolism of Jaspine B. Consistent with this observation, 80% of the administered Jaspine B was degraded after 1 h incubation with rat liver microsomes. In conclusion, the facilitated intestinal permeability in the presence of bile salts could significantly increase the bioavailability of Jaspine B and could lead to the development of oral formulations of Jaspine B with bile salts. Moreover, the highly distributed features of Jaspine B in the brain, kidney, heart, and spleen should be carefully considered in the therapeutic effect and toxicity of this compound.

## 1. Introduction

The development of anticancer drugs with effective therapeutic mechanisms is most interesting [1]. In that sense, various herbal and marine compounds have emerged as potential alternative medicines because of their structural diversity and safety, which was demonstrated by their long history of dietary use [2,3]. Marine natural products have been isolated since 1950s and marine sponges are one of the richest sources of bioactive compounds, showing the anti-proliferative effect through the inhibition of microtubule formation, the promotion of cell growth arrest, and stimulation of the cells death program [1]. Of these, the discovery of the sponge-derived nucleoside such as spongothymidine and spongouridine was made and three marine-based, anti-cancer drugs are currently being marketed [4]. Cytarabine was the first marine-derived anticancer drug approved in 1998 for the treatment of acute myelogenous leukemia. It was isolated from the marine sponge, Cryptotethya crypta, and induced apoptotic signals by inhibiting the NF-κB/Rel nuclear factor and binding to Bcl-2, and resulting in growth arrest at the G1/S phase [5]. Trabectedin, isolated from Ecteinascidia turbinata, was an approved anti-cancer drug against metastatic soft tissue carcinoma and ovarian cancer in 2009 in Europe [1]. Another approved marine drug is eribulin, an analog of halichondrin B, extracted from the marine sponge, Halichondria okadai [6], in 2010 from the FDA for the treatment of metastatic breast cancer. It also showed Bcl-2 inactivation and inhibition of microtubule polymerization [1,6].

Metabolites of sphingolipids such as ceramide, sphingosine, and sphingosine-1-phosphate (S1P), have been emerged as modulators for cancer progression [7]. Several studies addressing the effectiveness of sphingosine kinase SphK1 inhibition for cancer therapy have been reported [8,9,10,11]. The mRNA levels of SphK1 were significantly increased in breast, colon, lung, ovary, uterus, and kidney cancer patients, as well as in acute leukemia patients [12,13,14], and the downregulation of SphK1 decreased the epidermal growth factor and reduced prolactin- and E2-induced migration in metastatic breast cancer [7].

**Figure 1:**
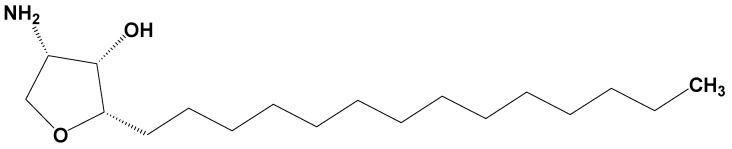
Structure of Jaspine B.

Jaspine B (pachastrissamine) (Figure 1) is an anhydrophytosphingosine, which is extracted from the marine sponge, *Pachastrissa* spp. [15]. It showed effective anti-cancer activity against several human carcinomas. Owing to its structural similarity with sphingosine, Jaspine B inactivated SphK1 and induced apoptotic signals [16]. In addition, Jaspine B inhibited melanoma cell growth by inhibiting the phosphorylation of Forkhead box O3 (FOXO3) [17] and by inducing apoptosis [18].

Previously, we had reported the anti-tumor activity of Jaspine B against various tumor cells that overexpressed sphingosine kinase. The results showed that Jaspine B was the most effective against breast cancer cells (MCF-7, IC_50_ = 2.31 μM) and showed differential cytotoxicity towards human breast adenocarcinoma (MDA-MB-231, IC_50_ > 100 μM), renal carcinoma (786-O, IC_50_ = 29.4 μM), melanoma (MDA-MB-435, IC_50_ = 2.60 μM), ovarian (SK-OV3, IC_50_ = 4.78 μM), and hepatoma (HepG2, IC_50_ = 5.69 μM) cells. The steady-state cellular concentration of Jaspine B was associated with the cytotoxic effect [19].

However, there have been few studies determining the absorption, disposition, and pharmacokinetic properties of Jaspine B, despite the importance of understanding these properties of the active, natural components. Therefore, in this study, we aimed to evaluate the pharmacokinetics, absorption, disposition, and excretion profile of Jaspine B and to investigate the underlying mechanisms related to its pharmacokinetic properties.

## 2. Results

### 2.1. LC/MS-MS Analysis of Jaspine B in the Biological Samples

**Figure 2:**
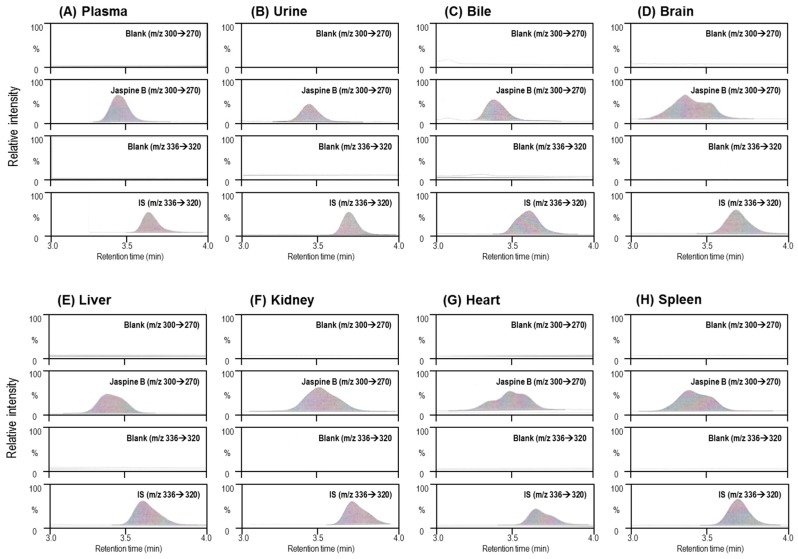
Representative multiple reaction monitoring (MRM) chromatograms of rat (**A**) plasma, (**B**) urine, (**C**) bile, and tissue homogenates of (**D**) brain, (**E**) liver, (**F**) kidney, (**G**) heart, and (**H**) spleen samples. In each panel from A to H, the chromatograms represent the MRM of Jaspine B (*m*/*z* 300→270) and internal standard (IS) (berberine, *m*/*z* 336→320) from blank and biological samples, respectively.

We developed an analytical method of Jaspine B in the rat plasma, urine, bile, and various tissue homogenates using a liquid chromatography tandem-mass spectrometry (LC-MS/MS) system. Figure 2A showed the representative multiple reaction monitoring (MRM) chromatograms obtained from the analysis of blank rat plasma and rat plasma samples at 1 h after the oral dose of 30 mg/kg Jaspine B. In addition, representative MRM chromatograms obtained from other resources such as urine, bile, and tissue homogenates of brain, kidney, liver, heart, and spleen were also shown in Figure 2B–H. Although the concentration range of Jaspine B varied depending on the biological samples used, the analyses of Jaspine B in other resources such as urine, bile, and tissue homogenates of brain, kidney, liver, heart, and spleen did not show any interference at the retention times of Jaspine B (Figure 2).

### 2.2. Pharmacokinetics of Jaspine B Following Intravenous Injection of 10 mg/kg Jaspine B

**Figure 3:**
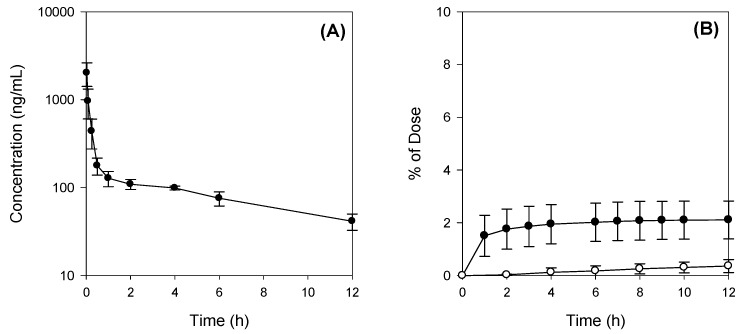
(**A**) Plasma concentration-time profile and (**B**) biliary (●) and urinary (○) excretion of Jaspine B after intravenous injection of Jaspine B (10 mg/kg) in rats. The means ± S.D. from four different rats.

The mean plasma concentration–time profiles of Jaspine B after IV administration at a dose of 10 mg/kg in rats are shown in Figure 3A and the relevant pharmacokinetic parameters are listed in Table 1. The plasma concentration of Jaspine B after a IV administration declined, with a bi-exponential elimination process, and the terminal half-life was calculated as 6.7 h. The distribution half-life, which was calculated from the distribution phase in Figure 3A, was 1.4 h, and the volume of distribution at steady-state was calculated as 21.2 L/kg, suggesting a fast distribution of this compound into the second compartment.

The excreted amounts of Jaspine B via the urinary and biliary routes are shown in Figure 3B. After 10 mg/kg of Jaspine B was administered, 0.4 ± 0.2% and 2.4 ± 0.8% of the IV dose were recovered in the urine and bile, respectively (Table 1), suggesting that there was substantial metabolism of Jaspine B during its disposition. This was also supported by the large systemic clearance (CL_total_) of Jaspine B, 98.3 mL/min/kg and low CL_bile_ and CL_urine_ of Jaspine B compared with CL_total_. The contribution of CL_bile_ and CL_urine_ was about 4% of CL_total_ (Table 1).

The cumulative excreted amounts of Jaspine B were measured for 72 h using a metabolic cage. After 10 mg/kg of Jaspine B was intravenously administered, 1.6 ± 0.7% and 4.7 ± 0.8% of the dose were recovered after 72 h from the urine and feces, respectively. Therefore, over the 72 h period, the recovery of unaltered Jaspine B was estimated to be 6.3% of the single IV dose from the combined feces and urine samples, suggesting that a large amount of Jaspine B was metabolized in vivo.

### 2.3. Metabolic Instability of Jaspine B

**Figure 4:**
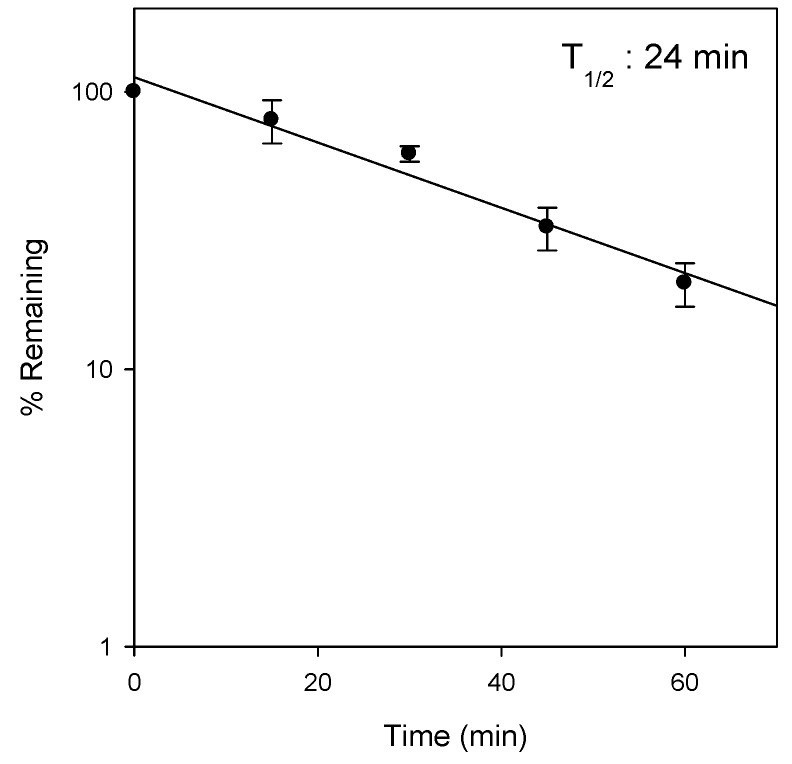
Metabolic instability of Jaspine B with rat liver microsomes. Jaspine B (1 μM) was incubated with 0.25 mg rat liver microsomes in the presence of a *β*-nicotinamide adenine dinucleotide 2′-phosphate reduced (NADPH)-generating system (1.3 mM *β*-NADP, 3.3 mM glucose-6-phosphate, 3.3 mM MgCl_2_, and 1.0 unit/mL glucose-6-phosphate dehydrogenase) for 60 min at 37 °C in a shaking water bath. The half-life (T_1/2_) was calculated from the first-order degradation rate constant.

Given the evidence of the substantial metabolism of Jaspine B, its microsomal stability was measured using rat liver microsomes. The percentage remaining after incubating for 30 min and 60 min was 59.8% and 20.4%, respectively, and the degradation half-life was calculated as 24 min (Figure 4). These results suggest the metabolic instability of Jaspine B in the liver, which is consistent with the previous results of the low contribution of biliary and urinary excretion of Jaspine B to the large clearance of this compound.

### 2.4. Bile Acid Facilitates the Intestinal Absorption of Jaspine B

**Figure 5:**
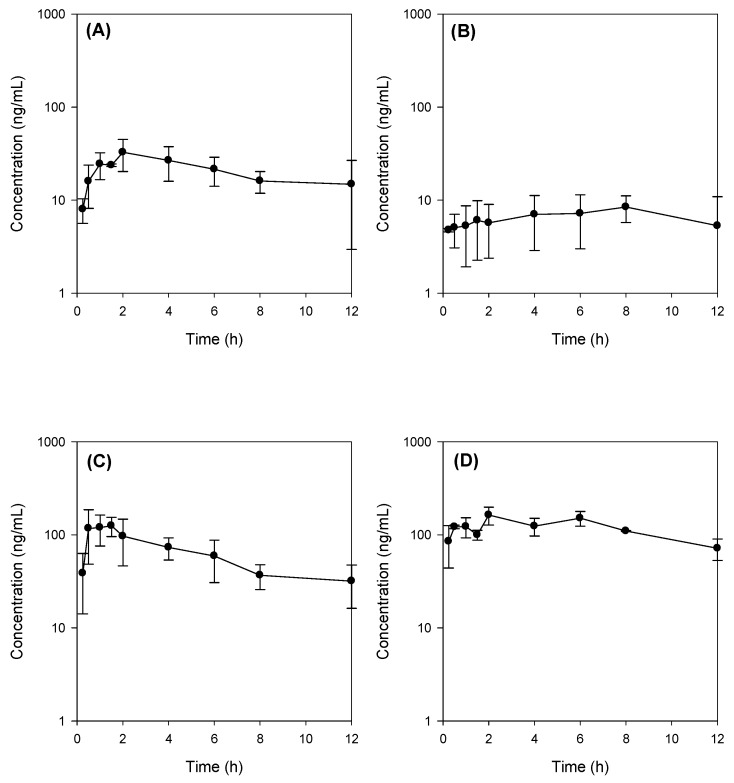
Plasma concentration-time profile of Jaspine B in rats after oral administration of Jaspine B (30 mg/kg) with or without taurocholate (TC; 60 mg/kg) in control and bile-depleted rats. (**A**) Control group; (**B**) bile-depleted group; (**C**) bile-depletion + TC; (**D**) Control + TC group. Data are expressed as the means ± S.D. from three to five different rats.

The mean plasma concentration–time profiles of Jaspine B after oral administration at a dose of 30 mg/kg in rats are shown in Figure 5A, and the relevant pharmacokinetic parameters are listed in control group (Table 2). Absolute bioavailability (BA), calculated by dividing the oral AUC by the intravenous AUC, was calculated as 6.2%.

Interestingly, the plasma concentration of Jaspine B decreased in the rats with bile depletion (Figure 5B) compared with that of the control group (Figure 5A). In our study, bile depletion was accomplished with 4 h drainage of bile through the bile cannula, which was cannulated to the bile duct using PE-10 tubing. After this bile drainage, the concentration of total bile salts in the bile sample decreased from 50.1 ± 6.5 mM to 15.3 ± 5.7 mM (i.e., 70% decrease), which was measured using an enzymatic-fluorometric assay with the slight modifications reported by Choi et al. [20].

However, the co-administration of taurocholate (TC, 60 mg/kg) with Jaspine B in bile-depleted rats prevented the decrease in plasma Jaspine B and elevated the levels above those of controls (Figure 5C). This was also observed when TC was administered to the control rats; in this situation, the plasma concentration of Jaspine B was higher than that of the control group (Figure 5D vs. Figure 5A). The addition of TC increased the C_max_ and AUC of orally administered Jaspine B without changing the terminal half-life (T_1/2_) (Table 2). These results suggest that the increase in C_max_ and AUC by the addition of TC was attributed to the absorption phase rather than the disposition phase, and consequently, the addition of TC increased the oral bioavailability of this compound from 6.2% to 41.2% (Table 2). In case of bile-depleted rats, AUC_∞_ and T_1/2_ were not calculated because the elimination rate constant could not be obtained. Therefore, the oral bioavailability of Jaspine B was calculated as AUC_12h,PO_/AUC_12h,IV_.

**Figure 6:**
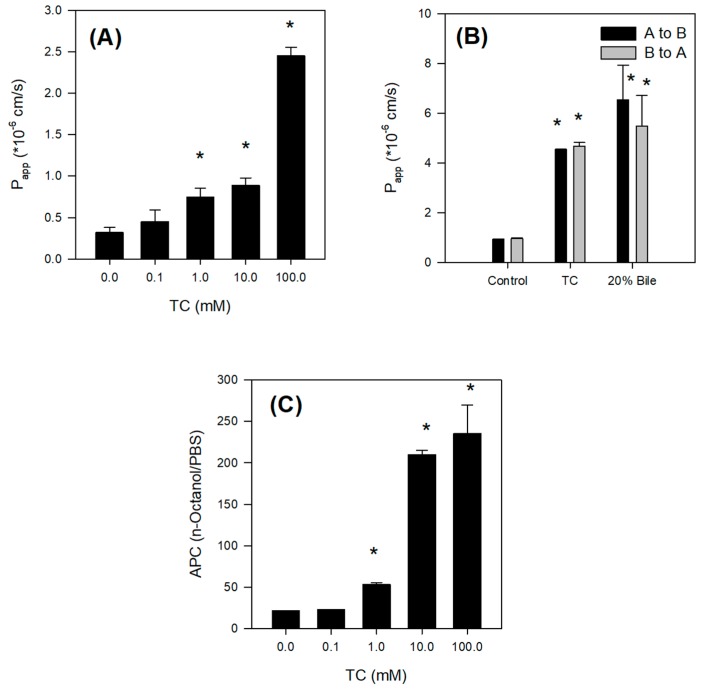
(**A**) Effect of taurocholate (TC) on the permeability of Lucifer yellow in the rat ileum. Permeability (P_app_) of Lucifer yellow (10 μM) in the presence of TC (0, 0.1, 1, 10, and 100 mM) was measured in the rat ileum using the Ussing chamber system. (**B**) Effect of TC and bile on the permeability of Jaspine B in the rat ileum. Permeability (P_app_) of Jaspine B (10 μM) in the presence of TC (10 mM) and bile (20%) was measured in the rat ileum using the Ussing chamber system. (**C**) Effect of TC on the lipophilicity of Jaspine B. Apparent partition coefficient (APC) of Jaspine B between *n*-octanol and PBS phases was measured in the presence of TC (0, 0.1, 1, 10, or 100 mM).Each data point represents the mean ± S.D. of three independent experiments. * *p* < 0.05, statistically significant versus the corresponding control group.

To confirm the role of bile salts in intestinal absorption, we measured intestinal permeability in rat intestinal segments using an Ussing chamber system. At first, to determine the optimized bile salt concentration, we monitored the permeability of Lucifer yellow, a marker compound for paracellular permeation and cell integrity [21,22], with various concentrations of TC (0.1–100 mM). The permeability of Lucifer yellow increased gradually until 10 mM TC and then showed a sharp increase at 100 mM TC (Figure 6A), suggesting that the cell integrity was disrupted between 10 and 100 mM TC.

To determine whether the intestinal absorption was increased by the addition of 10 mM TC, we measured the absorptive (A to B direction) and secretory (B to A direction) permeability of Jaspine B with 10 mM TC. The addition of 10 mM TC increased both absorptive and secretory permeability approximately 5-fold. The increased Jaspine B permeability (~5-fold) was greater than the increased Lucifer yellow permeability caused by tight junction opening (~2.8-fold), which was 56% of the increased Jaspine B permeability (Figure 6B). Therefore, the facilitated intestinal permeability in the presence of TC was caused partly by tight junction opening and partly by another unidentified mechanism. Since the absorptive and secretory permeabilities were almost identical, the intestinal transport mechanism such as efflux pump or uptake transport process may not contribute to the intestinal absorption of this compound [23]. The addition of 20% bile also increased the permeability of Jaspine B (Figure 6B) where the concentration of total bile salts in 20% bile was 10.1 ± 2.3 mM, which is similar to the concentration of bile salts in the intestinal lumen [24] as well as equilvalent to 10 mM TC. Thus the increased Jaspine B permeability in the presence of 10 mM TC and 20% bile was not statistically different.

Next, to unveil other mechanisms of increased Jaspine B permeability, we investigated whether the lipophilicity of Jaspine B increased in the presence of TC, since tertiary amines and quaternary ammonium can interact with endogenous bile to form lipophilic ion-pair complexes of Jaspine B and TC [25,26,27]. The partition coefficient of Jaspine B increased 10-fold in the presence of 10 mM TC compared with that in the absence of TC (Figure 6C).

Collectively, these results show that the enhancement of Jaspine B absorption in the presence of 20% bile and 10 mM TC could be attributed to the increased passive diffusion by the formation of lipophilic ion-pair complexes of Jaspine B-bile salts, as well as increased paracellular permeation through the tight junction opening by bile or bile salts [22,25]. As consequence, the increased absorption of Jaspine B in the presence of bile salts would lead to the increased BA of Jaspine B.

### 2.5. Tissue Distribution of Jaspine B

**Figure 7:**
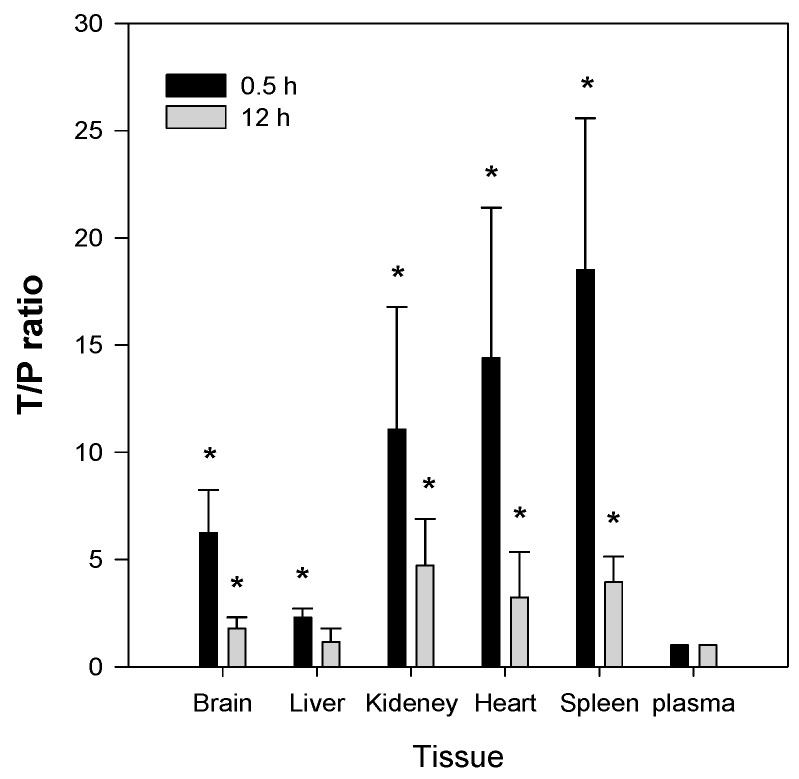
Tissue to plasma (T/P) ratios of Jaspine B recovered from tissues at 0.5 h and 12 h after intravenous (10 mg/kg) administration of Jaspine B to rats. At 0.5 h and 12 h after dosing, plasma, liver, kidney, heart, spleen, and brain samples were collected and the concentrations of Jaspine B in the samples were measured. The bars represent the means ± S.D. of the results from four rats. * *p* < 0.05, significant versus plasma ratio.

To investigate the tissue distribution of Jaspine B, its concentrations in the brain, liver, kidney, heart, spleen, and plasma were measured at 0.5 h and 12 h after IV administration of 10 mg/kg. The liver, kidney, heart, and spleen were selected as major organs that show high distribution, and the brain was selected as an organ that shows limited distribution. To calculate the drug concentration ratios of tissue to plasma (T/P ratio) of Jaspine B, 0.5 h and 12 h were selected to represent the distribution and elimination phases, respectively. As shown in Figure 7, the T/P ratios of Jaspine B in the brain, kidney, heart, and spleen were significantly higher than that in the plasma at 0.5 h (i.e., 6.2-, 11.1-, 14.4-, and 18.5-fold greater in the brain, kidney, heart, and spleen, respectively, than in the plasma). The significantly higher concentrations of Jaspine B in the brain, kidney, heart, and spleen were maintained 12 h after administration (i.e., 1.8-, 4.7-, 3.2-, and 3.9-fold greater in the brain, kidney, heart, and spleen, respectively, than in the plasma). These results were consistent with the large volume of distribution of Jaspine B, 21.2 L/kg. Interestingly, the concentration of Jaspine B in the liver was lower than in the other tissues (i.e., 2.3- and 1.2-fold higher than plasma levels at 0.5 and 12 h, respectively), which may be attributed to the substantial metabolism of this compound in the liver.

## 3. Discussion

Jaspine B is an anhydrophytosphingosine, extracted from the marine sponge, *Pachastrissa* spp. It shows meaningful inhibitory activity against sphingosine kinases (SphK1 and SphK2), which is an important mechanism of action for its anti-proliferative effect [28]. Jaspine B shows differential efficacy against a variety of tumor types of different tissue origins, and the cellular accumulation of Jaspine B at steady-state is a crucial determining factor of efficacy [19]. Moreover, the intravenous injection of Jaspine B (200 mg/mouse, equivalent to 6.7 mg/kg) on 4th, 8th, and 12th day dramatically decreased metastatic melanoma cell growth in the lungs of Jaspine B-injected mice on day 14 [18]. Although this in vivo and in vitro anti-proliferative effects of Jaspine B, the pharmacokinetics features of this compound was not investigated.

Therefore, the next step in understanding the anti-tumor activity of Jaspine B was to investigate its pharmacokinetic characteristics and the underlying mechanisms controlling the absorption, distribution, metabolism, and elimination of this compound.

The absorption of Jaspine B itself was limited, however, and the presence of bile salts increased the intestinal permeability of this compound over 5-fold by increasing partitioning of Jaspine B to lipophilic phase and tight junction opening (Figure 6). However, since the TC infusion to the rat liver did not significantly alter the metabolic activity of sulfobromophthalein [29], it is unlikely to conclude that concomitant administration of Jaspine B with TC inhibited metabolism of Jaspine B, and consequently increased BA of Jaspine B. To the contrary, the co-existence of Jaspine B with TC increased intestinal permeability and thereby increased the intestinal absorption and BA of Jaspine B. The results were consistent with a low oral BA of Jaspine B (6.2%) that decreased to 1.6% in bile-depleted rats, and after the co-addition of TC, oral BA increased to 23.5% in bile-depleted rats and 41.2% in control rats. Since the increased permeability of Jaspine B was the modulating factor that increased the low oral BA of Jaspine B and the permeability of Jaspine B in the rat intestine was very limited (0.11 × 10^−6^ cm/s), the important factors for the low BA of Jaspine B can be elucidated as low intestinal permeability. In addition, the low oral BA was known to be attributed to the limited permeability, the chemical and enzymatic degradation of a drug in the gut lumen, and the intestinal or hepatic first-pass effect [30]. The instability of Jaspine B in the gut lumen and the hepatic or intestinal first-pass effect also need to be investigated to elucidate the underlying mechanisms for the low BA of Jaspine B.

In our study, bile depletion was accomplished with 4 h drainage of bile through the bile cannula, which was evidenced by the 70% reduction of total bile salts concentration (from 50.1 ± 6.5 mM in control rats to 15.3 ± 5.7 mM) in bile drainage rats, which we named as bile-depleted rats. Since over 90% of bile salts were recirculated gut-liver-bile cycle [31], a 70% decrease of bile salts concentration in the bile implicated the significant decrease of bile salts in the gut as well, which resulted in the decreased absorption of Jaspine B in bile-depleted rats (Figure 5B). As a result of TC supplementation (60 mg/kg) with Jaspine B, which was suspended in 3 mL of DMSO: PEG400: saline solution, the TC concentration would be approximately 10 mM in the gut and could enhance the absorption of Jaspine B in rats from bile depletion + TC group and control + TC group (Figure 5C,D).

These results could be applied to the development of oral Jaspine B formulations consisting of bile salts and lipophilic phospholipids, i.e., mixed micelle formulations [32], to increase the permeability and lipophilicity of this compound. However, for the clinical and pharmaceutical application, the use of pharmaceutical excipients that enhance the transport without affecting tight junctions of intestine rather than high concentrations of TC would be the best approach. For example, SP1049C (Supratek Pharma Inc., Montreal, PQ, Canada), a doxorubicin-containing mixed-micelle formulation with Pluronic L61 and F127, showed increased cellular concentration of the doxorubicin in tumor cells and has reached clinical phase 3 study because of its superior antitumor activity compared with that of doxorubicin standard formulation [33]. A phospholipid-Tween 80 mixed micelle formulation of paclitaxel showed higher anti-tumor activity and reduced systemic toxicity than Taxol formulation did [34].

The second feature of Jaspine B is that it is highly distributed to tissues such as brain, kidney, heart, and spleen. The T/P ratios in these organs were 6.2, 11.1, 14.4, and 18.5, respectively, and remained elevated 2–4-fold 12 h after a single IV administration of Jaspine B (Figure 7). Since we previously reported that the steady-state drug concentration was important for the anti-cancer efficacy of this compound [19,35,36], the tissue distribution of Jaspine B is very important to predict the therapeutic efficacy of this compound. Moreover, this compound showed differential cytotoxicity depending on the cancer cell type, thus the highly tissue distributed features of this compound should be carefully considered in terms of drug response and toxicity. However, we should note that the use of PEG400 as a vehicle in the tissue distribution study (Figure 7) might change the tissue distribution profile of Jaspine B because PEG400 has an ability to entrap of this compound in the spleen’s reticuloendothelial systems and thereby increase the in vivo metabolic stability compared with in vitro metabolic stability results (Figure 4).

In addition, the low the recovery from feces and urine (about 6.3% after IV administration) was consistent with the observed microsomal instability in the rat liver microsomes (Figure 4), although the involvement CYP enzymes as well as the proposed metabolite structures were not investigated in this study. Therefore, identification of metabolites and the elucidation of the metabolic enzyme(s) involved will be necessary for future investigation. Moreover, since the metabolites of Jaspine B may have pharmacological activities on the inhibition of SphK1 and the inhibition of FOXO3 phosphorylaiton, the evaluation of the pharmacological activity or toxicity of the proposed metabolite would be of importance.

## 4. Materials and Methods

### 4.1. Reagents

Jaspine B was synthesized by Dr. D. Lee (Ajou University, Suwon, Korea) with a purity of over 99% and was confirmed by nuclear magnetic resonance (NMR) and mass spectroscopy results [37]. Its structure is shown in Figure 1.

Hank’s balanced salt solution (HBSS), taurocholate (TC), Lucifer yellow, dimethyl sulfoxide (DMSO), polyethylene glycol (PEG) 400, *β*-nicotinamide adenine dinucleotide (*β*-NAD), 3α-hydroxysteroid dehyrogenase (3α-HSD), EDTA, and Tris base were purchased from Sigma-Aldrich (St. Louis, MO, USA). Fetal bovine serum, Dulbecco’s Modified Eagle’s medium (DMEM), and penicillin-streptomycin were purchased from Hyclone Laboratories (Logan, UT, USA). All other reagents were reagent grade.

Rat liver microsomes (RLMs) from Sprague Dawley (SD) rats and the reaction solutions, including the NADH-generating system were purchased from BD-Corning (Corning, NY, USA).

A NaviCyte Ussing chamber system was obtained from Harvard Apparatus Co. (Holliston, MA, USA). Round metabolic cages for rats were purchased from Jungdo B&P Inc. (Seoul, Korea).

### 4.2. LC-MS/MS Analysis of Jaspine B

The concentrations of Jaspine B in the biological samples were analyzed using an Agilent 6430 Triple Quad LC/MS-MS system (Agilent, Wilmington, DE, USA) coupled with an Agilent Infinity 1290 series high performance liquid chromatography (HPLC) system. The separation was performed on a Synergi Polar RP column (2.0 mm i.d.× 150 mm, 4 μm, Phenomenex) using a mobile phase that consisted of acetonitrile and DDW (85:15, *v*/*v*) with 0.1% formic acid at a flow rate of 0.2 mL/min. Mass spectra were recorded by electrospray ionization in the positive mode. Quantification was performed using multiple reaction monitoring (MRM) at *m*/*z* 300.3→270.2 for Jaspine B and *m*/*z* 336.1→320.0 for berberine.

For the analytical validation of Jaspine B in plasma samples, the standard curve range was 25–5000 ng/mL and the concentrations of quality control (QC) samples were 75, 500, and 3000 ng/mL. The recovery of these spiked QC sample were in the range of 88.2–98.1%. Intra- and inter-day precision and accuracy had coefficients of variance of less than 10%. The stability of Jaspine B QC samples after 3 freeze thaw cycles were 83.9–94.4% and the short-term stability of these samples after standing for 6 h at room temperature was 94.3–100.1%.

The respective standard curve range and sample preparation methods in various biological samples from various experiments were described in detail in each experimental section.

### 4.3. Pharmacokinetics of Jaspine B

Male SD rats (aged 8–9 weeks, weighing 250–300 g) were obtained from Samtako Bio Korea, Inc. (Osan, Korea). The rats were acclimatized for 1 week in a temperature-controlled room (23 ± 2 °C) with a 12-h illumination cycle. Food and water were given *ad libitum*. All animal procedures were approved by the Animal Care and Use Committee of the Kyungpook National University (No. 2015-0064). The rats were fasted for at least 12 h before the oral administration of drugs.

The femoral artery, femoral vein, and bile duct were cannulated with polyethylene tubes (PE-50 and PE-10; Jungdo, Seoul, Korea) under light anesthesia with isoflurane.

For intravenous (IV) dosing, Jaspine B was dissolved in a vehicle containing DMSO: PEG400: saline (2:4:4, *v*/*v*/*v*) and was IV-injected via the femoral vein at 10 mg/kg (vehicle volume, 1 mL/kg). Blood samples were collected from the femoral artery at 0, 0.083, 0.167, 0.25, 0.5, 1, 2, 4, 6, 8, and 12 h following the IV administration of Jaspine B. Bile and urine samples were collected every 2 h for a total of 12 h. After centrifugation of blood samples at 13,200 rpm for 10 min, aliquots of 50 μL of plasma, bile, and urine were stored at −80 °C until the analysis of Jaspine B.

In the experiment using metabolic cages, feces were collected at 12, 24, 48, and 72 h following the IV administration of Jaspine B (10 mg/kg). Aliquots of 50 μL of urine and 10% feces homogenates were stored at −80 °C until the analysis.

Aliquots (50 μL) of plasma, bile, feces homogenates, and urine samples were added to 250 μL of acetonitrile containing 0.5 ng/mL of berberine (an internal standard). After vortexing for 10 min and centrifugation at 13,200 rpm for 5 min, an aliquot (2 μL) of the supernatant was injected directly into the LC-MS/MS system. For the analysis of plasma concentration, the standard curve range was 25–5000 ng/mL and the concentrations of quality control (QC) samples were 75, 500, and 3000 ng/mL, as described previously. Concentrations of Jaspine B in urine, bile, and feces homogenates were 5–300 ng/mL, 20–5000 ng/mL, and 20–5000 ng/mL, respectively. The concentrations of QC samples were 15, 100, and 300 ng/mL for urine samples and 75, 500, and 3000 ng/mL for bile and feces homogenates.

For oral dosing, Jaspine B was dissolved in a vehicle containing DMSO: PEG400: saline (2:4:4, *v*/*v*/*v*) and was administered by oral gavages at a single dose of 30 mg/kg of Jaspine B concomitantly with or without 60 mg/kg TC (vehicle volume, 3 mL/kg) to the control and bile-depleted rats. Bile depletion was accomplished with 4 h drainage of bile through the bile cannula, which was cannulated to the bile duct using PE-10 tubing.

Blood samples (approximately 250 μL each) were collected from the femoral artery at 0, 0.25, 0.5, 1, 1.5, 2, 4, 8, and 12 h following the oral administration of Jaspine B. After centrifugation of blood samples at 13,200 rpm for 10 min, plasma samples (50 μL) were collected and stored at −80 °C until the analysis by liquid chromatography tandem-mass spectrometry (LC-MS/MS). Aliquots (50 μL) of plasma samples were added to 250 μL of acetonitrile containing 0.5 ng/mL of berberine (an internal standard). After vortexing for 10 min and centrifugation at 13,200 rpm for 5 min, an aliquot (10 μL) of the supernatant was injected directly into the LC-MS/MS system. The standard curve range was 5–2000 ng/mL and the concentrations of quality control (QC) samples were 60, 300, and 2000 ng/mL. Standard curves showed good linearity (R^2^ > 0.999) and the interday accuracy and precision was 99.0–105.1% and 0.3–6.9%, respectively.

### 4.4. Tissue Distribution of Jaspine B

Jaspine B was dissolved in a vehicle containing DMSO: PEG400: saline (2:4:4, *v*/*v*/*v*) and IV-injected via the femoral vein at a dose of 10 mg/kg (vehicle volume, 1 mL/kg). Blood samples were collected from the abdominal artery and animals were euthanized at 0.5 h and 12 h after IV dosing. The blood, brain, liver, kidney, heart, and spleen were immediately excised, gently washed with ice-cold saline, and weighed. The tissue samples were homogenized with 9 volumes of saline. Aliquots of 50 μL of tissue homogenates and plasma were stored at −80 °C until the analysis.

Aliquots (50 μL) of plasma and tissue homogenates were added to 250 μL of acetonitrile containing 0.5 ng/mL of berberine. After vortexing for 10 min and centrifugation at 13,200 rpm for 5 min, an aliquot (2 μL) of the supernatant was injected directly into the LC-MS/MS system. For the analysis of plasma and tissue concentration, the standard curves with a range of 5–1000 ng/mL in the blank plasma and 10% bank tissue homogenates from brain, liver, kidney, heart, and spleen were prepared, and the concentrations of quality control (QC) samples of respective plasma and tissue homogenates were 25 and 750 ng/mL. The recovery of these spiked QC samples were in the range of 73.6–112.2% in all tissue homogenates. Intra- and inter-day precision and accuracy had coefficients of variance of less than 15%.

### 4.5. Metabolic Stability of Jaspine B in Rat Liver Microsomes (RLMs)

Jaspine B (1 μM) was reconstituted in 100 mM potassium phosphate buffer (pH 7.4) containing 0.25 mg of RLMs and was pre-incubated for 5 min at 37 °C. The reaction was initiated by adding an NADPH-generating system (1.3 mM *β*-NADP, 3.3 mM glucose-6-phosphate, 3.3 mM MgCl_2_, and 1.0 unit/mL glucose-6-phosphate dehydrogenase) and then incubated (final volume 100 μL) for 0, 15, 30, and 60 min at 37 °C in a shaking water bath. The reaction was stopped by placing the incubation tubes on ice and adding 200 μL of ice-cold acetonitrile containing 0.5 ng/mL of berberine. After vortexing for 10 min and centrifugation at 13,200 rpm for 5 min, 2 μL of the supernatant was injected directly into the LC-MS/MS system. We also determined the metabolic stability of 1 μM propranolol and 1 μM metformin as a positive and negative control, respectively, using the same procedure [38]. The remaining % of propranolol and metformin after 60 min incubation was 12.2% and 63.4% of initial concentration, indicating the feasibility of this system.

### 4.6. Determination of Bile Salts Concentration in Bile

Total bile salt concentrations in bile samples were determined by means of an enzymatic-fluorometric assay with the slight modification of Choi at al. [20]. Briefly, bile samples were collected. Fifty μL aliquots of standard TC solutions (5, 10, 25, 50, 100, 200 μM), bile samples were added to 950 μL of reaction buffer containing 1mM *β*-nicotinamide adenine dinucleotide (*β*-NAD), 50 μU 3*α*-hydroxysteroid dehyrogenase (3*α*-HSD), 0.385 mM EDTA and 760 mM Tris (pH 9.5), followed by incubation at 37 °C for 30 min. The reaction was quenched by the addition of 3 mL of ice cold water and the fluorescence was measured at an excitation wavelength of 340 nm and an emission wavelength of 465 nm.

### 4.7. Determination of the Intestinal Permeability of Jaspine B

For the measurement of the effect of TC on the tight junction of biological membranes, the permeability of 50 μM Lucifer yellow, a marker of paracellular permeation [21], was measured in the presence of TC (0, 0.1, 1, 10, 100 mM). Prior to the experiment, ileal segments from SD rats (about 20 cm) were placed in the chambers and were submerged in fresh, prewarmed (37 °C) HBSS for 15 min for acclimatization. The chambers were continuously bubbled with carbogen gas (5% CO_2_/ 95% O_2_) during the experiment. The experiments began by replacing the HBSS with HBSS containing 50 μM Lucifer yellow and various concentrations of TC on the apical side (A) and adding fresh HBSS to the basal side (B). Aliquots (400 μL) of media were withdrawn from the receiver compartment (B) after 0, 20, 40, 60, and 80 min, and the volume of liquid in the receiver compartment was replenished with fresh, prewarmed HBSS after each sampling. Aliquots (200 μL) of all samples were used for the determination of Lucifer yellow concentration. The fluorescence of Lucifer yellow in the samples was measured using a fluorescence spectrophotometer (Infinite 200 PRO, Tecan, Switzerland) with excitation at 425 nm and emission at 535 nm.

A to B and B to A permeability of Jaspine B was measured in the presence of TC and rat bile. One mL of HBSS containing 50 μM Jaspine B and 10 mM TC or 20% bile was added to the donor side and 1 mL of preheated fresh HBSS was added to the receiver side. Aliquots (400 μL) of media were withdrawn at 0, 20, 40, 60, and 80 min from the receiver compartment as described above. Aliquots (50 μL) of all samples were stored at −80 °C until analysis.

Aliquots (50 μL) of all samples were added to 250 μL of acetonitrile containing 0.5 ng/mL of berberine. After vortexing for 10 min and centrifugation at 13,200 rpm for 5 min, 2 μL of the supernatant was injected directly into the LC-MS/MS system.

### 4.8. Effect of TC on the Lipophilicity of Jaspine B

The effect of TC on the apparent partition coefficients (APC) of Jaspine B between aqueous and organic phases was investigated [26]. *n*-Octanol and phosphate-buffered saline (PBS, pH 7.4) were used as the organic and aqueous phases, respectively, in the partition study. *n*-Octanol and PBS were saturated with respect to one another prior to use. An equal volume of *n*-octanol was added to PBS (5 mL each) containing Jaspine B (10 mM) and TC (0, 0.1, 1, 10, or 100 mM) in a screw-capped test tube, and the mixture was vortexed vigorously for 5 min followed by shaking for 2 h at 25 °C in a temperature-controlled water bath. After standing for 1 h at 25 °C in the water bath, the mixture was separated into two phases by centrifugation at 3000 rpm for 10 min. The concentration of Jaspine B in a 50-μL aliquot of aqueous and organic phase was measured. Briefly, 50 μL of sample was added to 250 μL of acetonitrile containing 0.5 ng/mL of berberine. After vortexing for 10 min and centrifugation at 13,200 rpm for 5 min, an aliquot (2 μL) of the supernatant was injected directly into the LC-MS/MS system. The APC was calculated from the concentration ratio of each compound between the octanol and PBS phases.

### 4.9. Data Analysis

Pharmacokinetic parameters were determined using a non-compartmental analysis (WinNonlin^®^ 2.0; Pharsight, Mountain View, CA, USA). Maximum plasma concentration (C_max_) and time to reach C_max_ (T_max_) values were obtained from plasma concentration-time curves. The area under the plasma concentration–time curve from zero to infinity (AUC) was calculated using the trapezoidal-extrapolation method. For extrapolation, the area from the last data point to infinity was estimated by dividing the terminal-phase rate constant by plasma concentration at the last time point. Oral bioavailability was calculated by dividing AUC_PO_, which was normalized by Jaspine B dose (30 mg/kg) by AUC_IV_, which was also normalized by IV dose of Jaspine B (10 mg/kg):

(1) $$\mathrm{BA}(\%)=\frac{\frac{{\mathrm{AUC}}_{\infty ,\mathrm{PO}}}{\mathrm{Dose}(30\mathrm{mg}/\mathrm{kg})}}{\frac{{\mathrm{AUC}}_{\infty ,\mathrm{IV}}}{\mathrm{Dose}(10\mathrm{mg}/\mathrm{kg})}}\times 100$$

However, in the case of bile-depleted rats, AUC_∞_ and T_1/2_ were not calculated because the elimination rate constant could not be obtained. Therefore, the oral bioavailability of Jaspine B was calculated as follows:

(2) $$\mathrm{BA}(\%)=\frac{\frac{{\mathrm{AUC}}_{12h,\mathrm{PO}}}{\mathrm{Dose}(30\mathrm{mg}/\mathrm{kg})}}{\frac{{\mathrm{AUC}}_{12h,\mathrm{IV}}}{\mathrm{Dose}(10\mathrm{mg}/\mathrm{kg})}}\times 100$$

Total systemic clearance (CL_total_) was calculated by dividing IV dose by plasma AUC and the apparent volume of distribution at steady-state (Vss) was calculated by multiplying mean residence time (MRT) by CL_total_. Urinary and biliary clearances of Jaspine B (CL_urine_ and CL_bile_) were estimated by dividing the total amount of Jaspine B excreted into urine and bile, respectively, for the 0–12 h period by plasma AUC.

The apparent permeability (P_app_) of the drug was calculated by dividing the initial drug transport rate (dQ/dt, pmol/min) by the initial drug concentration in the donor compartment of the insert (C_o_) multiplied by the surface area of the insert (A, cm^2^) [22]:

(3) $$P_{\mathrm{app}}=\frac{\mathrm{dQ}}{\mathrm{dt}}\times \frac{1}{{\mathrm{AC}}_{o}}$$

Data are expressed as the means ± standard deviations (S.D.), and statistical significance was determined by *t*-test using SPSS software version 16.1.

## Figures and Tables

**Table 1:** Pharmacokinetic parameters of Jaspine B after intravenous injection of Jaspine B at a dose of 10 mg/kg in rats.

Parameters		IV (10 mg/kg)
Plasma	T_1/2_ (h)	6.7 ± 1.6
	MRT (h)	3.6 ± 0.5
	AUC_12h_ (ng∙h/mL)	1286.9 ± 165.6
	AUC_∞_ (ng∙h/mL)	1701.9 ± 137.1
	Vss (L/kg)	21.2 ± 2.6
	CL_total_ (mL/min/kg)	98.3 ± 7.7
Bile	X_bile_ (% of dose)	2.4 ± 0.8
	CL_bile_ (mL/min/kg)	3.1 ± 0.6
Urine	X_urine_ (% of dose)	0.4 ± 0.2
	CL_urine_ (mL/min/kg)	0.5 ± 0.3

Data are expressed as the means ± S.D. from four different rats.

**Table 2:** Pharmacokinetic parameters of Jaspine B after oral administration of Jaspine B at a dose of 30 mg/kg in rats.

Parameters	Control (*n* = 3)	Bile Depletion (*n* = 3)	Bile Depletion + TC (*n* = 5)	Control + TC (*n* = 3)
C_max_ (ng/mL)	36.3 ± 11.3	8.5 ± 2.8 *	163.4 ± 28.4 *	174.1 ± 13.0 *
T_max_ (h)	2.5 ± 1.3	9.3 ± 2.3 *	1.0 ± 0.5	3.3 ± 2.3
T_1/2_ (h)	5.5 ± 1.1	−	7.2 ± 1.5	6.4 ± 2.8
MRT (h)	4.9 ± 0.4	6.1 ± 0.3 *	4.9 ± 0.5	5.5 ± 0.03
AUC_12h_ (ng∙h/mL)	240.6 ± 57.8	80.3 ± 42.7 *	828.3 ± 180 *	1406.3 ± 4.3 *^,#^
AUC_∞_ (ng∙h/mL)	314.4 ± 76.1	−	1201.7 ± 254 *	2100.9 ± 419 *^,#^
BA (%)	6.2	1.6	23.5	41.2

Each value represents the mean ± S.D. * *p* < 0.05, significant versus control group; ^#^ *p* < 0.05, significant versus Bile depletion + TC group.
